# Use of a multi-phased approach to identify and address facilitators and barriers to the implementation of a population-wide genomic screening program

**DOI:** 10.1186/s43058-023-00500-9

**Published:** 2023-10-11

**Authors:** Caitlin G. Allen, Katherine Sterba, Samantha Norman, Amy Jackson, Kelly J. Hunt, Lori McMahon, Daniel P. Judge

**Affiliations:** 1 Department of Public Health Science, College of Medicine, Medical University of South Carolina, Charleston, SC USA; 2 In Our DNA SC, Medical University of South Carolina, Charleston, SC USA; 3 Research Office, Medical University of South Carolina, Charleston, SC USA

## Abstract

**Introduction:**

Population-wide genomic screening for CDC Tier-1 conditions offers the ability to identify the 1–2% of the US population at increased risk for Hereditary Breast and Ovarian Cancer, Lynch Syndrome, and Familial Hypercholesterolemia. Implementation of population-wide screening programs is highly complex, requiring engagement of diverse collaborators and implementation teams. Implementation science offers tools to promote integration of these programs through the identification of determinants of success and strategies to address potential barriers.

**Methods:**

Prior to launching the program, we conducted a pre-implementation survey to assess anticipated barriers and facilitators to reach, effectiveness, adoption, implementation, and maintenance (RE-AIM), among 51 work group members (phase 1). During the first year of program implementation, we completed coding of 40 work group meetings guided by the Consolidated Framework for Implementation Research (CFIR) (phase 2). We matched the top barriers to implementation strategies identified during phase 2 using the CFIR-ERIC (Expert Recommendation for Implementing Change) matching tool.

**Results:**

Staffing and workload concerns were listed as the top barrier in the pre-implementation phase of the program. Top barriers during implementation included adaptability (*n* = 8, 20%), complexity (*n* = 14, 35%), patient needs and resources (*n* = 9, 22.5%), compatibility (*n* = 11, 27.5%), and self-efficacy (*n* = 9, 22.5%). We identified 16 potential implementation strategies across six ERIC clusters to address these barriers and operationalized these strategies for our specific setting and program needs.

**Conclusion:**

Our findings provide an example of successful use of the CFIR-ERIC tool to guide implementation of a population-wide genomic screening program.

**Supplementary Information:**

The online version contains supplementary material available at 10.1186/s43058-023-00500-9.

Contributions to the literature

- This study contributes to the literature by applying the Reach, Effectiveness, Adoption, Implementation, and Maintenance (RE-AIM) framework and the Consolidated Framework for Implementation Research (CFIR) to identify barriers and facilitators in the pre-implementation planning phase and the implementation phase for a population-wide genomic screening program.
- CFIR and the Expert Recommendations for Implementing Change (ERIC) frameworks were then used to understand the determinants of success and identify strategies to address potential barriers in the implementation of programs.
- The study identifies key facilitators to implementation, including trialability, cosmopolitanism, networks and communication, leadership engagement, and availability of resources. These findings contribute to the literature by highlighting strategies that can assist with implementing population-wide genomic screening programs.
- The study identifies key barriers to implementation, including adaptability, complexity, patient needs and resources, compatibility, and self-efficacy. These findings contribute to the literature by highlighting specific challenges that need to be addressed when implementing population-wide genomic screening programs.
- The study provides operationalized strategies, derived from the CFIR-ERIC matching tool, to address the identified barriers in the specific setting and program needs. These strategies offer practical insights for other programs seeking to implement similar population-wide genomic screening initiatives, enhancing the literature with actionable recommendations for successful implementation.

## Introduction

Centers for Disease Control and Prevention’s (CDC) Tier 1 genomic conditions of Hereditary Breast and Ovarian Cancer Syndrome, Lynch Syndrome, and Familial Hypercholesterolemia affect 1–2% of the US population [1–4]. Individuals who are identified with a pathogenic or likely pathogenic variant for genes associated with these conditions are at a substantially elevated risk of serious, yet avoidable disease. Poor identification of individuals at higher risk for CDC Tier 1 conditions represents a missed opportunity to enhance public health as early detection and intervention could significantly reduce morbidity and mortality [5]. New population-based approaches to identification of individuals with predisposition to these conditions have been recommended by the National Academies of Science, Engineering, and Medicine’s Genomic Action Collaborative. In 2018, this group endorsed population-based genomic screening for nine genes associated with CDC Tier 1 conditions [5]. Given the growing interest in population-based approaches to identifying individuals with susceptibility to CDC Tier 1 conditions and rapidly expanding number of programs offering genomic screening, it is essential to understand how to strengthen the implementation of this approach.

As a field, implementation science offers the tools to guide and promote the implementation of population-based screening programs among diverse populations and settings. The Consolidated Framework for Implementation Research (CFIR), for example, is a widely used and well-operationalized determinants framework that is designed to identify barriers and facilitators during different phases of implementation [6]. Prior literature has identified multilevel barriers and facilitators to implementing population-based screening, including individual level psychosocial and attitudinal barriers (e.g., anxiety, worry about screening, negative emotional impact) and perceived lack of clinical utility among providers (i.e., does not add benefit to current practice) [7]. Community and health system barriers included concern related to confidentiality, privacy, and impact of genetic results on insurance coverage.

To date, efforts have largely been limited to identification of barriers and facilitators, with a strong focus on perceptions of individuals when deciding whether to participate in the population-based genomic screening programs, as opposed to implementation barriers and facilitators [7]. While it is valuable to address individual participant’s perceptions regarding participation in the program, the practical aspects of implementing population-based screening programs are understudied. Furthermore, the field of implementation science has emphasized the importance of identifying how to strategically address implementation barriers and facilitators once identified. Implementation strategies are designed to improve implementation and help address identified barriers, with perhaps the most well-known compilation of implementation strategies being the Expert Recommendations for Implementing Change (ERIC) [8]. This compilation of 73 discrete implementation strategies was designed to help researchers and implementers make plans to overcome existing or anticipated barriers to implementation. The 73 strategies are further grouped into nine thematic clusters using concept mapping, which allows for recognition of broader themes. In 2019, the CFIR-ERIC matching tool was published to link identified CFIR barriers to ERIC implementation strategies and advance the implementation of new initiatives [9, 10]. The use of implementation science frameworks can help support our understanding of how to optimally implement population-based screening in health systems.

The objective of this study was to (1) describe perceived pre-implementation barriers and facilitators to delivery of a population-wide genomic screening initiative before program launch, (2) describe real world implementation barriers and facilitators during program delivery, and (3) apply the CFIR-ERIC matching tool to identify how these barriers and facilitators can be addressed to enhance population-wide genomic screening outcomes.

## Methods

### Description of population wide genomic screening program

The In Our DNA SC population-wide genomic screening program was launched at the Medical University of South Carolina (MUSC) in November 2021 in partnership with Helix, a leading population genomics company. This program offers free genomic screening to 100,000 individuals for CDC Tier 1 conditions. As a research study, individuals consent to participate and then provide a saliva sample for processing. Results are returned to the participant’s medical records. Those who are identified as positive for a CDC Tier 1 condition receive a phone call from study staff to disclose their results and are offered a free consultation with a genetic counselor. Details about the program have been previously published [11, 12].

### Pre-implementation barriers and facilitators survey (phase 1)

Prior to the launch of In Our DNA SC, in the first phase of this study, we surveyed individuals who were part of work groups responsible for implementing the program and providing support in the planning phase. The web-based, self-administered surveys were distributed via REDCap to work group members that included: administrative, clinical services and results, data technology and integration, evaluation and implementation research, marketing and communication, operations and staff training, and research enablement staff. The survey included eight 5-point Likert-scale questions aligned with RE-AIM outcomes (Reach, Effectiveness, Adoption, Implementation, and Maintenance) to evaluate work group member’s perceptions about translatability and dimensions that may be most relevant to real-world implementation [13]. Respondents were also asked to select whether each of the following were considered a barrier or facilitator to program success: administrative requirements, collaboration and teamwork, communication, education and training, financial resources, leadership support, staffing and workload, time as outlined by Li et al. [14]. And the survey included space for qualitative feedback about perceived pre-implementation barriers and facilitators to launching In Our DNA SC (see Supplemental Material A). Institutional Review Board approval was not required for distribution of this survey because these activities were considered quality improvement rather than research.

Descriptive statistics were used (mean, standard deviation; frequency, percent) to report pre-implementation work group barriers and facilitators and implementation barriers and facilitators.

### Implementation barriers and facilitators (phase 2)

We used the CFIR framework during phase 2 of this study to code experienced barriers and facilitators reported during work group meetings that occurred during the implementation phase of the program (see Supplemental Material B). In total, there were 40 meetings that took place between November 2021 and November 2022. We developed a CFIR tracking log using a REDCap form. This log included space for a study team member to document notes about the meeting and code barriers and facilitators mentioned during the meeting in real time. The CFIR tracking log included all CFIR domains and constructs, with coding taking place at the construct level. Coding was completed by one trained coder with experience in CFIR per meeting. All logs were quality checked by the first author after the meeting and discrepancies were revolved through discussion between the coder and the first author.

To complete the CFIR-ERIC matching, we used the publicly available CFIR-ERIC Implementation Strategy Mapping tool [15, 16]. This tool was developed to help match strategies to CFIR barriers identified by study teams. Seventy-three implementation strategies are included in the tool, derived from the ERIC list of strategies [15, 17]. Implementation strategies are provided based on “cumulative percent” or the strength of endorsement for that strategy. The CFIR-ERIC matching tool provides level 1 endorsed ERIC strategies and level 2 endorsed strategies. Level 1 strategics indicate that more than 50% of the experts included in the original CFIR-ERIC tool development ranked the ERIC strategy as one of their top seven strategies for that barrier. Level 2 endorsed ERIC strategies indicate that between 20 and 50% of experts ranked this as one of their top seven strategies to address the specific barrier [15].

As described in the tool, we selected the top barriers identified during implementation of In Our DNA SC (adaptability, complexity, patient needs and resources, compatibility, and self-efficacy). We selected these barriers because they were identified during at least 20% of the work group meetings throughout the implementation phase. Once we entered these barriers, the CFIR-ERIC tool provided output linking ERIC strategies mapped onto each of the barriers and a cumulative percent of which strategies mapped. We report the ERIC strategies with at least a 20% cumulative matching percentage. We aligned these ERIC strategies with one of nine ERIC clusters [9]. To complete the CFIR-ERIC mapping, we tailored new implementation strategies or resources for our program and program-specific target audiences to those ERIC strategies with greater than 75% cumulative percentage using the Proctor recommendations for specifying the reporting implementation strategies [16]. The final implementation strategies were reviewed by a panel of seven implementation and evaluation experts from MUSC and Helix during a regular implementation and evaluation team meeting to ensure identified ERIC strategies were appropriately tailored to population-wide genomic screening as well as our specific setting and program.

## Results

### Pre-implementation barriers and facilitators (phase 1)

A total of 51 implementation work group members responded to the pre-implementation barriers and facilitators survey. This included individuals from across workgroups: clinical services and results (*n* = 16, 31.4%), data technology and integration (*n* = 8, 15.7%), evaluation and implementation research (*n* = 3, 5.9%), marketing and communication (*n* = 8, 15.7%), operations and staff training (*n* = 1, 2%), and research enablement (*n* = 15, 29.4%).

Work group members expressed concern about the ability to reach a representative group of participants (M = 2.95, SD = 0.99) but felt that it would be possible to overcome barriers to reaching the target population (M = 3.29, SD = 0.84). Specifically, individuals indicated in the qualitative components of the survey the importance of ensuring that MUSC addresses historical injustices and transgressions related to inappropriate use of minorities in research, mistrust, the need for ethical oversight, and to ensure diverse groups are included (Reach). Effectiveness was assessed based on whether the In Our DNA SC program was perceived to lead to the anticipated outcomes of delivering actionable genetic results to MUSC patients (M = 3.56, SD = 0.95) and that it would provide lasting benefits to the participants (M = 3.54, SD = 0.99) (Effectiveness). Respondents indicated average levels of confidence in the ability for sites and staff to adopt the In Our DNA SC program (M = 3.48, SD = 0.85) (Adoption). When assessing implementation, the work group members overall felt that the program could be delivered as intended (M = 3.34, SD = 0.92) and could be delivered by individuals representing a variety of positions and levels of expertise (M = 3.35, SD = 0.97). In the qualitative component of the survey, individuals indicated that the rapid timeline of go-live required that leadership reduce team’s other work requirements to hyper-focus on successful implementation (Implementation). Finally, the implementation team stated that they believed the program could be maintained over time (M = 3.42, SD = 0.84) (Maintenance) (Table 1).

**Table 1:** RE-AIM pre-implementation perceptions among work group members (*n* = 51)

		**Mean**	**SD**
Reach	How confident are you that In Our DNA SC will successfully attract all members of the target population regardless of age, race/ethnicity, gender, and socioeconomic status?	2.95	0.99
	Rate how confident you are that you can overcome barriers to reaching the target population	3.29	0.84
Effectiveness	Rate your confidence that In Our DNA SC will lead to the planned outcome of delivering actionable genetic risk insights to MUSC patients	3.56	0.95
	How confident are you that In Our DNA SC will produce lasting benefits for participants?	3.54	0.99
Adoption	How confident are you that In Our DNA SC will be adopted by sites and staff that are part of this phase of the program?	3.48	0.85
Implementation	How confident are you that In Our DNA SC can be consistently delivered as intended?	3.34	0.92
	How confident are you that In Our DNA SC can be delivered by staff and providers representing a variety of positions, levels and expertise?	3.35	0.97
Maintenance	How confident are you that In Our DNA SC will be sustained after it has been implemented?	3.42	0.84

Scale range 1–4 with higher numbers reflecting higher outcomes

**Fig. 1:**
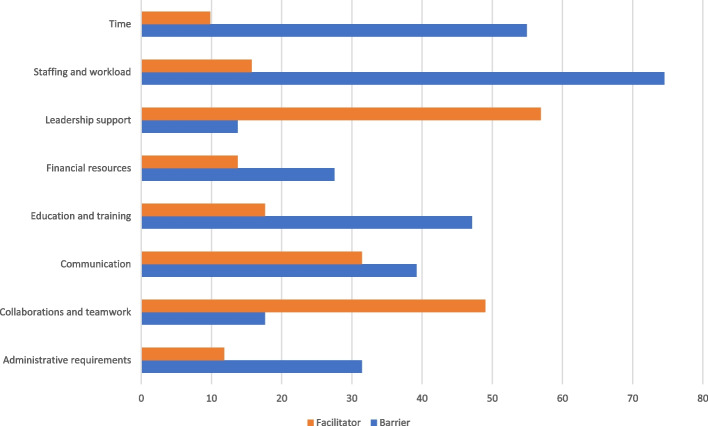
Pre-implementation barriers and facilitators identified by work group members (*N* = 51)

The top-rated pre-implementation barriers identified among the work groups included staffing and workload (*n* = 38, 74.5%) and time (*n* = 28, 54.9%). The top-rated pre-implementation facilitators among work group members included communications and teamwork (*n* = 25, 49%) and leadership support (*n* = 29, 56.9%) (Fig. 1).

### Implementation barriers and facilitators (phase 2)

We report CFIR barriers and facilitators from forty work group meetings that took place between November 2021 and November 2022 (Table 2) during the implementation period. Adaptability (*n* = 8, 20%) and complexity (*n* = 14, 35%) were the top-rated intervention characteristic barriers. Patient needs and resources (*n* = 9, 22.5%) was the top-rated outer setting barrier. Specifically, we found challenges with addressing interests of existing MUSC patients without upcoming clinical appointments, a need to educate uninsured community members about their options, and challenges with program participants receiving genetic counseling from a South Carolina genetic counselor. Compatibility (*n* = 11, 27.5%) was the top-rated inner setting barrier, with top challenges being the compatibility of the In Our DNA SC technological needs with MUSC’s electronic health record system. Finally, self-efficacy was the top-rated characteristics of individuals barrier (*n* = 9, 22.5%), with the barrier primarily focused on ensuring that clinical staff feel comfortable with sample collection workflow for patients.

**Table 2:** CFIR barriers and facilitators during implementation period (*n* = 40)

	**Facilitator**		**Barrier**	
	***n***	**%**	***n***	**%**
**Intervention characteristics**				
Intervention source	3	7.5	2	5
Evidence, strength, and quality	2	5	0	0
Relative advantage	0	0	0	0
Adaptability	21	52.5	8	20
Trialability	9	22.5	2	5
Complexity	2	5	14	35
Design, quality, and packaging	8	20	5	12.5
Cost	3	7.5	6	15
**Outer setting**				
Patient needs and resources	10	25	9	22.5
Cosmopolitanism	8	20	2	5
Peer pressure	3	7.5	1	2.5
External policies and incentives	4	10	6	15
**Inner setting**				
Structural characteristics	3	7.5	6	15
Networks and communication	19	47.5	7	17.5
Culture	1	2.5	1	2.5
**Inner setting—implementation climate**				
Tension for change	5	12.5	5	12.5
Compatibility	4	10	11	27.5
Relative priority	10	25	1	2.5
Organizational incentives and rewards	1	2.5	0	0
Goals and feedback	6	15	2	5
Learning climate	6	15	1	2.5
**Inner setting—readiness for implementation**				
Leadership engagement	16	40	5	12.5
Availability of resources	11	27.5	7	17.5
Access to knowledge and information	2	5	4	10
**Characteristics of individuals**				
Knowledge and beliefs about intervention	5	12.5	6	15
Self-efficacy	6	15	9	22.5
Individual stage of change	0	0	0	0

Barriers and facilitators were assessed in real time by project staff in workgroup meetings (*N* = 40) during the implementation period using a CFIR-guided tracking form

Facilitators identified through the implementation work group logs included adaptability (*n* = 21, 52.5%) and trialability (*n* = 9, 22.5%) as intervention characteristics. The program was able to develop new messaging (adaptability) throughout the implementation of the program and test new messages for recruitment with staff prior to disseminating (trialability). Outer setting facilitators included patient needs and resources (*n* = 10, 25%) and cosmopolitanism (*n* = 8, 20%). To ensure patient needs and resources were met, the team expanded services to more clinics for better outreach and spread to communities. Inner setting facilitators included networks and communication (*n* = 19, 47.5%), relative priority of In Our DNA SC for MUSC (*n* = 10, 25%), leadership engagement (*n* = 16, 40%), and availability of resources (*n* = 11, 27.5%).

### Matching CFIR-ERIC strategies and identifying implementation strategies

Our top five barriers included adaptability, complexity, patient needs and resources, compatibility, and self-efficacy. We used the CFIR-ERIC matching tool to identify implementation strategies to address these barriers (Table 3). We identified 43 potential ERIC implementation strategies across nine clusters (use evaluation and iterative strategies, provide interactive assistance, adapt and tailor to context, develop collaborator interrelationships, train and educate collaborators, support clinicians, engage consumers, use financial strategies, change infrastructure) [9]. Twenty-seven of these strategies (62.7%) were considered level 1 with a cumulative percentage greater than or equal to 50% and sixteen were considered level 2 (37.2%). One level 1 strategy was identified (73% match) to promote adaptability (cluster: adapt and tailor to content). No level 1 matches occurred for the barrier of complexity. Three level 1 matches were found for the barrier of patient needs and resources: (1) involve patients/consumers and family members (cluster: engage consumers, 71% match), (2) conduct local needs assessment (cluster: use evaluative and iterative strategies, 57% match), and (3) obtain and use patient/consumer and family feedback (cluster: use evaluative and iterative strategies, 76% match). No level 1 matches were found for barriers of compatibility and self-efficacy.

**Table 3:** Recommended ERIC strategies mapped to CFIR implementation barriers

ERIC Cluster	ERIC Strategies		Primary Endorsed CFIR Barriers				
		Cumulative Percent	Adaptability	Complexity	Patient Needs & Resources	Compatibility	Self-efficacy
Adapt and tailor to content	Promote adaptability	**183%**	**73%**	*40%*	14%	*45%*	11%
Adapt and tailor to content	Tailor strategies	**125%**	*35%*	*27%*	14%	*38%*	11%
Develop collaborator interrelationships	Identify and prepare champions	**108%**	*23%*	*30%*	5%	*21%*	*30%*
	Conduct local consensus discussions	**107%**	*31%*	7%	*29%*	*41%*	0%
	Capture and share local knowledge	**103%**	*35%*	*27%*	10%	14%	19%
	Model and simulate change	**83%**	19%	*27%*	0%	3%	*33%*
	Identify early adopters	**76%**	*27%*	*20%*	0%	10%	19%
	Organize clinician implementation team meetings	**53%**	8%	*20%*	0%	14%	11%
	Build a coalition	**50%**	15%	0%	14%	*21%*	0%
	Visit other sites	*48%*	19%	3%	0%	10%	15%
	Use advisory boards and workgroups	*43%*	4%	0%	*29%*	3%	7%
	Use an implementation adviser	*40%*	8%	10%	5%	10%	7%
	Inform local opinion leaders	*36%*	15%	13%	0%	3%	4%
Engage consumers	Involve patients/consumers and family members	**93%**	8%	0%	**71%**	10%	4%
	Prepare patients/consumers to be active participants	**51%**	0%	0%	*48%*	3%	0%
	Intervene with patients/consumers to enhance uptake & adherence	*38%*	8%	3%	*24%*	3%	0%
Provide interactive assistance	Facilitation	**93%**	*27%*	*20%*	0%	*24%*	*22%*
	Provide local technical assistance	**61%**	4%	17%	5%	14%	*22%*
	Provide clinical supervision	*33%*	0%	7%	5%	10%	11%
	Centralize technical assistance	*31%*	0%	10%	0%	10%	11%
Support clinicians	Facilitate relay of clinical data to providers	*28%*	4%	3%	10%	3%	7%
	Create new clinical teams	*27%*	0%	3%	10%	7%	7%
Train and educate collaborators	Create a learning collaborative	**100%**	*23%*	*33%*	0%	14%	*30%*
	Conduct ongoing training	**77%**	0%	*37%*	0%	0%	*41%*
	Provide ongoing consultation	**77%**	8%	*20%*	5%	3%	*41%*
	Conduct educational meetings	**60%**	12%	13%	10%	10%	15%
	Develop educational materials	**56%**	12%	13%	10%	3%	19%
	Shadow other experts	**55%**	12%	7%	0%	3%	*33%*
	Make training dynamic	**54%**	0%	10%	0%	3%	*41%*
	Conduct educational outreach visits	*45%*	12%	7%	5%	0%	*22%*
	Distribute educational materials	*23%*	12%	3%	5%	0%	4%
	Use train the trainer strategies	*21%*	0%	7%	0%	0%	15%
Use evaluative and iterative strategies	Assess for readiness and identify barriers and facilitators	**140%**	*31%*	*30%*	*33%*	*34%*	11%
	Conduct cyclical small tests of change	**133%**	*23%*	*37%*	10%	*38%*	*26%*
	Conduct local needs assessment	**116%**	*35%*	3%	**57%**	*21%*	0%
	Obtain and use patients/consumers and family feedback	**94%**	4%	0%	**76%**	10%	4%
	Develop a formal implementation blueprint	**70%**	8%	*43%*	5%	3%	11%
	Purposely reexamine the implementation	**61%**	12%	17%	5%	*28%*	0%
	Stage implementation scale up	**55%**	0%	*30%*	0%	10%	15%
	Audit and provide feedback	*41%*	4%	3%	5%	7%	*22%*
	Develop and implement tools for quality monitoring	*28%*	0%	7%	14%	3%	4%
	Develop and organize quality monitoring systems	*25%*	4%	10%	0%	3%	7%
Utilize financial strategies	Alter incentive/allowance structures	*30%*	0%	7%	10%	10%	4%

Only included strategies >20% matc

Tool is designed to match strategies to barriers identified using CFIR

**Bold numbers** = Level 1 endorsements according to CFIR-ERIC Matching tool

*Italic numbers* = Level 2 endorsements according to CFIR-ERIC Matching tool

Cumulative Percentage= strength of endorsement for that strategy across all CFIR barriers

Choosing implementation strategies to address contextual barriers: diversity in recommendations and future directions | Implementation Science | Full Text (biomedcentral.com)

Table 4 shows ERIC strategies with a cumulative percentage match greater than 75%. We identified 16 potential implementation strategies across six ERIC clusters to address barriers. The clusters included: adapt and tailor content (strategies of: promote adaptability, tailor strategies), develop collaborator interrelationships (strategies of: identify and prepare champions, conduct local consensus discussions, capture and share local knowledge, model and stimulate change, identify early adopters), engage consumers (strategies of: involve patients/consumers and family members), provide interactive assistance (strategies of: facilitation), train and educate collaborators (strategies of: create a learning collaborative, conduct ongoing training, provide ongoing consultation), and use evaluative and iterative strategies (strategics of: assess for readiness and identify barriers and facilitators, conduct cyclical small tests of change, conduct local needs assessment, and obtain and use patient/consumer and family feedback). Three co-authors (CA, KS, SN) worked to tailor these recommended strategies to our specific program goals using the Proctor recommendations for specifying and reporting implementation strategies (name it, define it, specify it) [16]. We presented these strategies to the implementation and evaluation research work group during a regular group meeting to help ensure that the strategies were aligned with our specific program. Consensus about the description of these strategies was achieved with group discussion among the implementation and evaluation research work group.

**Table 4:** Resources and implementation strategies to address barriers

ERIC cluster	ERIC strategies	Cumulative percent	New implementation strategy or resource	Target audience
Adapt and tailor to content	Promote adaptability	**183%**	Promote adaptability through team-directed program modifications to improve fit (e.g., adding a pictograph to the consent form, adding at home for sample collection) Marketing team developed a toolkit to guide tailored strategies for different clinics/patients/communities to improve reach Marketing team tailor materials to emphasize specific health benefits and education on how participants can benefit from prevention measures to enhance reach	Providers Participants Community Groups
	Tailor strategies	**125%**		
Develop collaborator interrelationships	Identify and prepare champions	**108%**	Study team protocolized stepped approach of relationship-building with identified key messengers at each step (e.g., meetings with clinic director, slide deck for meetings to identify tailored workflows, individualized champion MyChart messages to potential participants) to identify and prepare champions to improve effectiveness and implementation Marketing team incorporates messaging and marketing from leadership that highlights compatibility of program with other broader initiatives at institution to improve implementation and maintenance Marketing team identified and showcased stories of early adopters to enhance reach	Participants Implementation Teams
	Conduct local consensus discussions	**107%**		
	Capture and share local knowledge	**103%**		
	Model and simulate change	**83%**		
	Identify early adopters	**76%**		
Engage consumers	Involve patients/consumers and family members	**93%**	Study team facilitated a community advisory board (CAB) provide guidance, participant testimonials to enhance representativeness and reach Study team encourages participants to engage with website prior to singing up to enhance participant understanding of the program and program reach	Participants
Provide interactive assistance	Facilitation	**93%**	Study team offers assistance via telephone or email for both patients and different clinical staff members to enhance adoption and effectiveness Study team provides coordinator training of new collection site staff members to build staff readiness to enhance adoption and effectiveness Study team coordinators are available to provide training to new staff as needed to enhance adoption and effectiveness	Participants Clinical Staff
Train and educate collaborators	Create a learning collaborative	**100%**	Study team conducted ongoing training by offering lunch and learns, provide updates on program status, community outreach toolkit to enhance maintenance	Researchers Providers Program Leadership
	Conduct ongoing training	**77%**		
	Provide ongoing consultation	**77%**		
Use evaluative and iterative strategies	Assess for readiness and identify barriers and facilitators	**140%**	Implementation and evaluation team conduct pre-implementation surveys with work group members and clinical site champions and staff to assess for readiness and identify barriers and facilitators Implementation and evaluation team conducted cyclical small test by starting with a pilot phase and expanding to other MUSC locations to improve reach and implementation Implementation team and marketing received patient/consumer feedback through the CAB guidance, workgroup meetings, and tracking marketing activities to improve reach	Implementation Teams Participants
	Conduct cyclical small tests of change	**133%**		
	Conduct local needs assessment	**116%**		
	Obtain and use patients/consumers and family feedback	**94%**		

## Discussion

To enhance the implementation of population-based genomic screening and application of implementation science to precision health, we first characterized barriers and facilitators to implementing a population-wide genomic screening program to guide the planning phase. We then identified barriers and facilitators experienced in the implementation period and matched these with ERIC implementation strategies.

The top barriers anticipated in the planning phase included staffing, workload, and time concerns while the top facilitators included communication and teamwork support. The assessment of potential barriers and facilitators of program success in the planning period can help guide program messaging, information dissemination channels, and team development. Identification of these anticipated barriers was helpful in informing the planning of the program, especially as program messaging, information dissemination, and teams were developed to support the program.

Moving into the implementation period, the top five barriers included adaptability, complexity, patient needs and resources, compatibility, and self-efficacy. These were spread across all CFIR domains. These results differ somewhat from prior research conducted by the Implementing GeNomics In PracTicE (IGNITE) Network. The IGNITE network used CFIR to conduct an analysis of six projects focused on genomic medicine and prioritized constructs across CFIR domains. IGNITE identified challenges to implementation and lessons learned across integration of genomic medicine at six sites in their network [18–20]. These included the following: improving relative advantage, strengthening self-efficacy and knowledge among clinicians, and engaging patients [20]. We similarly identified relative advantage as a key factor in our program; however, it was a considered a facilitator in the implementation of our program (*n* = 10, 25%), as were other implementation climate factors (leadership engagement (*n* = 16, 40%) and availability of resources (*n* = 11, 27.5%). IGNITE addressed relative priority through use of data warehousing techniques and prioritizing integration of genomics into the electronic health records. Another review of population screening implementation indicated that lack of integration between genomic data and electronic health records as a critical barrier to implementation [21]. From the beginning, our program worked closely with the information solutions and biomedical informatics leadership and teams to ensure integration, reducing this as a barrier to our implementation.

Similar to the IGNITE results, we found self-efficacy and knowledge to be a barrier to implementation and through the CFIR-ERIC matching tool are aligning planning to continue deploying educational materials, education, and outreach among clinicians. Finally, engaging patients (patient needs and resources) was identified in both IGNITE and the present study as a barrier. A systematic review about implementation of population genomic screening programs further emphasizes the importance of knowing patient needs and resources. To date, the literature has identified substantial intrapersonal barriers (psychosocial factors, knowledge, attitudes, and beliefs) that may limit likelihood of individuals to participate in population screening programs [7, 22, 23]. Successful implementation of population screening programs requires implementation strategies that address the needs of participants. As identified in IGNITE and the CFIR-ERIC tool, we are incorporating mass media, involving patients in implementation of the program through the community advisory board, and preparing patients to be active in their decisions.

Notably, adaptability, defined as the degree to which an intervention can be adapted, tailored, refined, or reinvented to meet local needs was coded a barrier as well as the top facilitator to implementation (*n* = 21, 52.5%) [6]. Adaptability was considered a facilitator throughout the duration of the implementation period but began being coded as a barrier to implementation in January 2023. While we did not conduct a formal time-focused analysis, the first 3 months of the program were framed as a “pilot phase” where the implementation teams were highly encouraged to provide feedback and make recommendations about modifications to the project. During the pilot phase, we identified 10 adaptations, with the majority designed to increase the number and type of patients contacted [24]. After these 3 months, adaptations continued, but the program was no longer considered to be in a pilot phase. Deeper exploration into the types of adaptations made to the program, intent of adaptations, and when adaptations occurred could help address the shift in the team’s perception of the program being highly adaptable in the early phases to less adaptable in later phase implementation [25].

Three of the barriers (complexity, compatibility, and self-efficacy) did not have a level 1 match using the CFIR-ERIC strategy matching tool. In the CFIR-ERIC matching tool, none of the ERIC strategies reached the appropriate level of consensus to be considered level 1. Complexity (perceived difficulty in intervention, reflected by the duration, scope, radicalness, disruptiveness, centrality, intricacy, and number of steps required to implement) has 15 level 2 strategies, compatibility (how well fits within workflows, systems, and processes) has 10 level 2 strategies, and self-efficacy (individual belief in ability to achieve implementation goals) has 12 level 2 strategies. In the original report describing the CFIR-ERIC mapping tool, authors indicated that a single strategy may simultaneously address multiple barriers or could have multiple pathways in producing positive implementation outcomes, depending on how it is operationalized. While the tool should provide guidance for the implementation strategies selected, it is critical that the tool is used aligned with the specific needs of the program being implemented. Clarity and specificity in the identified barriers are required to appropriately identify and the discrete implementation strategy to address that barrier. Indeed, our findings are the first to provide specificity to how to operationalize implementation strategies for population-wide genomic screening. Notably, we specified our strategies based on recommendations for strategy specification, but we did not further specify to different populations within our program. Because our program tests all individuals who enroll in for all CDC Tier 1 conditions (HBOC, LS, and FH), we chose not to further specify implementation strategies by population; however, future research could assess whether there are different determinants of implementation based on the population and thus more discrete implementation strategies.

Future work will assess the impact of the CFIR-ERIC implementation strategies identified on RE-AIM outcomes. RE-AIM outcomes will be measured in two ways: (1) through a comparison of work group member’s perceptions of the RE-AIM outcomes pre-implementation (as described in this paper) and in later phases of implementation and (2) quantitative measures of the impact of the implementation strategies on the RE-AIM measures identified by the program [26]. In a recent review, only 14% of manuscripts examined implementation outcomes in relation to implementation strategies (e.g., what is the impact of the implementation strategy on outcomes). Our future work will be able to assess both the impact of the strategies on implementation team member’s perceptions of the program and actual outcomes [27]. We identified 15 implementation strategies across the five main barriers. Deciding which strategies to prioritize and which workgroups to work with is a critical next step. We plan to take a practical approach and focus on implementation strategies that are most endorsed by the study team and leadership [28, 29]. Endorsement is currently completed informally through group discussion and consensus building, prioritizing implementation strategies that are both feasible and appropriate for the project. Other potential approaches to prioritizing implementation strategies could include mapping underlying mechanism of change [30]. We will continue to track when new implementation strategies are tested, and adaptations are made to the program and identify the impact on RE-AIM outcomes.

In addition to assessing implementation strategies and being responsive to the barriers identified, given the long-term, complex nature of the intervention, it is important to consider ways to continue building upon identified facilitators to encourage a spirit of innovation and adaptability beyond initial implementation. Capitalizing on identified facilitators (trialability, patients’ needs and resources, cosmopolitanism, relative priority, leadership engagement, and availability of resources) can support ongoing program optimization and success. Prior literature has found building implementation capacity and implementation infrastructure for a project or specific topic can support scale up and maintenance. We continue to build implementation capacity through expansion of our program at new clinical sites within our institution, hiring additional staff, and community partnerships. Infrastructure has also been built through clarifying implementation team roles, formalization of study procedures, and technical infrastructure within our electronic health record to support the project.

This study is not without limitations. In phase 1, we used RE-AIM to identify pre-implementation barriers and facilitators and contextual determinants that may impact successful program implementation among work group members. In phase 2, CFIR was used to code implementation barriers and facilitators identified during work group meetings based on the different goals for each phase of this research. We considered pre-implementation and implementation two distinct phases of our program; however, future work could focus on using a consistent framework to assess pre-implementation and implementation barriers and facilitators. This would allow for a more comprehensive overview of facilitators and barriers and allow for matching ERIC strategies across implementation phases. Our approach also relied on perceptions of barriers and facilitators (survey and coding of meetings) and not actual behaviors. We focused on identifying CFIR barriers using the original CFIR framework. Since beginning tracking, the new CFIR 2.0 has been published [31]. The updated CFIR 2.0 framework provides opportunities to better incorporate behavior change to understand barriers and facilitators to implementation. We plan to modify our tracking log to incorporate the new CFIR 2.0 constructs [31]. In addition, we focused on mapping CFIR barriers to ERIC implementation strategies. To date, the literature has predominately focused on barriers-implementation strategy mapping; however, there are potential opportunities to map facilitators to ERIC implementation strategies that focus on enhancing existing facilitators (e.g., capitalizing on facilitators or adding resources to facilitators). For example, we identified networks and communication (*n* = 19, 47.5%) and leadership engagement (*n* = 16, 40%) as two highly rated facilitators. The team could employ ERIC strategies to further enhance these as facilitators and continue to support the programs. Future work could also include qualitative interviews and/or focus groups to better understand pre-implementation and implementation barriers and facilitators.

## Conclusions

We engaged implementation teams and tracked barriers and facilitators through the pre-implementation and implementation phases of a population-wide genomic screening program. Through identification of the implementation barriers, we were able to use the CFIR-ERIC matching tool to prioritize how to address top-rated barriers. Additionally, we focused on tailoring the recommended ERIC strategies to our specific setting and program needs. Future work will track the impact of these implementation strategies on our program’s RE-AIM outcomes. As population wide genomic screening continues to become mainstream, identification of common facilitators and barriers to implementation, development of effective implementation strategies, and evaluating the effectiveness of these strategies will be critical [22]. Our findings advance the field of implementation science, as we offer more detailed and nuanced alignment of the CFIR-ERIC tool and apply this framework to a precision public health intervention.

### Supplementary Information

**Additional file 1: Supplemental Material A.** Check-in Survey Questions.

**Additional file 2: Supplemental Material B.** Data Dictionary Codebook.

## Data Availability

Data are available upon request from the corresponding author.
