# Supplementary material for: Use of a multi-phased approach to identify and address facilitators and barriers to the implementation of a population-wide genomic screening program
Source: Implement Sci Commun. 2023 Oct 11;4:122. doi: 10.1186/s43058-023-00500-9 (PMC10566189; doi:10.1186/s43058-023-00500-9)
Supplement: Supplementary file 1 — Additional file 1: Supplemental Material A. Check-in Survey Questions. [file 43058_2023_500_MOESM1_ESM.docx]

## CHECK-IN SURVEY QUESTIONS

Please respond to the questions below about your experience as part of the team that is implementing *In Our DNA SC.* Note that these questions are designed for responses as the program stands at the time of the survey.

1. What work group you are part of?

- Marketing and communication
- Data, technology, and integration
- Operations and staff training
- Research enablement
- Clinical services and results
- Administrative
- Evaluation and implementation research

1. What is your current role on the project?

- Work group leader
- Work group member

1. Which organization are you part of?

- MUSC
- Helix

### Adoption

1. How confident are you that *In Our DNA SC* will be adopted by settings and staff that are part of this phase of the program?
   - Not confident at all (1)
   - Not very confident (2)
   - Neither (3)
   - Fairly confident (4)
   - Very confident (5)

### Reach

1. How confident are you that *In Our DNA SC* will successfully attract all members of your target population regardless of age, race/ethnicity, gender, socioeconomic status and other important characteristics such as health literacy?
   - Not confident at all (1)
   - Not very confident (2)
   - Neither (3)
   - Fairly confident (4)
   - Very confident (5)
2. Rate how confident you are that you can overcome barriers to reaching the target population
   - Not confident at all (1)
   - Not very confident (2)
   - Neither (3)
   - Fairly confident (4)
   - Very confident (5)

### Implementation

1. How confident are you that *In Our DNA SC* can be consistently delivered as intended?

- Not confident at all (1)
- Not very confident (2)
- Neither (3)
- Fairly confident (4)
- Very confident (5)

1. How confident are you that *In Our DNA SC* can be delivered by staff and providers representing a variety of positions, levels and expertise?
   - Not confident at all (1)
   - Not very confident (2)
   - Neither (3)
   - Fairly confident (4)
   - Very confident (5)

### Effectiveness

1. Rate your confidence that *In Our DNA SC* will lead to the planned outcome of delivering actional genetic risk insights

- Not confident at all (1)
- Not very confident (2)
- Neither (3)
- Fairly confident (4)
- Very confident (5)

### Maintenance

1. How confident are you that *In Our DNA SC* will produce lasting benefits for participants?
   - Not confident at all (1)
   - Not very confident (2)
   - Neither (3)
   - Fairly confident (4)
   - Very confident (5)
2. How confident are you that *In Our DNA SC* will be sustained after it has been implemented?
   - Not confident at all (1)
   - Not very confident (2)
   - Neither (3)
   - Fairly confident (4)
   - Very confident (5)

## Barriers and Facilitators

1. Select any of the following that are current barriers to preparing for the launch of this phase of In Our DNA SC
   1. Administrative requirements
   2. Collaborations and teamwork
   3. Communication
   4. Education and training
   5. Financial resources
   6. Leadership support
   7. Staffing and workload
   8. Time
2. Select any of the following that are current facilitators to preparing for the launch of this phase of In Our DNA SC
   1. Administrative requirements
   2. Collaborations and teamwork
   3. Communication
   4. Education and training
   5. Financial resources
   6. Leadership support
   7. Staffing and workload
   8. Time
3. Please provide any additional feedback. What has been going well? What concerns do you have?

**References**

[Organizational contextual features that influence the implementation of evidence-based practices across healthcare settings: a systematic integrative review (nih.gov)](https://www.ncbi.nlm.nih.gov/pmc/articles/PMC5936626/)
