# Supplementary material for: Use of a multi-phased approach to identify and address facilitators and barriers to the implementation of a population-wide genomic screening program
Source: Implement Sci Commun. 2023 Oct 11;4:122. doi: 10.1186/s43058-023-00500-9 (PMC10566189; doi:10.1186/s43058-023-00500-9)
Supplement: Supplementary file 2 — Additional file 2: Supplemental Material B. Data Dictionary Codebook. [file 43058_2023_500_MOESM2_ESM.pdf]

Data Dictionary Codebook

08/31/2023 6:56am

#	Variable / Field Name	Field Label<br>*Field Note*	Field Attributes (Field Type, Validation, Choices, Calculations, etc.)
Instrument: **Work Group Meeting Logs** (work_group_meeting_logs) Enabled as survey			
1	[record_id]	Record ID	text
2	[name_of_work_group]	Name of Work Group	radio<br>1 Marketing and communication<br>2 Data, technology, and integration (BMIC and IS work group)<br>3 Operations and staff training<br>4 Research enablement<br>5 Clinical services and results (return of results)<br>6 Administrative<br>7 Evaluation and implementation research<br>8 Cross-functional leadership<br>9 Other (ad-hoc, add in notes)<br>10 Study Team Touch Base (added 12/1/21)<br>11 Clinical Reuse Planning (added 12/1/21)<br>12 Joint Oversight Committee (added 12/1/21)<br>13 Community Advisory Board
3	[date_of_meeting_mm_dd_yy]	Date of Meeting (MM/DD/YY)	text (date_mdy)
4	[topic_of_meeting]	Topic of Meeting	notes
5	[notes_barrier]	Section Header: *Implementation Barriers*<br>Implementation Barrier Notes	notes
6	[intervention_barrier]	Intervention Characteristics (BARRIERS)	checkbox<br>1 intervention_barrier___1 Intervention sour (whether In Our D was internal/exte developed)<br>2 intervention_barrier___2 Evidence, strength quality (whether I DNAS C was built good evidence)<br>3 intervention_barrier___3 Relative advantag Our DNA SC is bet than alternative s<br>4 intervention_barrier___4 Adaptability (In Ou SC can be

#	Variable	Field Label	Field Type	Code	Variable / Choice	Choice Label
						adapted/tailored/ discussions about adaptations that a needed or made)
				5	intervention_barrier___5	Trialability (can te Our DNA SC on sr scale)
				6	intervention_barrier___6	Complexity (how c radical, disruptive implementation is
				7	intervention_barrier___7	Design, quality, ar packaging (excelle how bundled, pre assembled)
				8	intervention_barrier___8	Cost (investment, opportunity)
7	[outersetting_barrier]	Outer Setting (BARRIERS)	checkbox			
				1	outersetting_barrier___1	Patient needs and resources (patients needs are accurately known and prioritized by organization)
				2	outersetting_barrier___2	Cosmopolitanism (network with other external universities/health systems)
				3	outersetting_barrier___3	Peer pressure (pressure to implement program based or other key peer organizations)
				4	outersetting_barrier___4	External policies and incentives (policy and regulatory mandates, collaboratives)
8	[innersetting_barrier]	Inner Setting (BARRIERS)	checkbox			
				1	innersetting_barrier___1	Structural characteristics (age, size, maturity of organization)
				2	innersetting_barrier___2	Networks and communication
				3	innersetting_barrier___3	Culture (norms, values, basic assumptions)
9	[inner_setting_impleme ntati]	Inner Setting - Implementation Climate (BARRIERS)	checkbox			

			1 | inner_setting_implementati___1 | Tension fo change (cu situation n changed- c about nee change an in the life c of implement<br>2 | inner_setting_implementati___2 | Compatibi between interventic existing systems)<br>3 | inner_setting_implementati___3 | Relative Pr (importanc implement within organizatic<br>4 | inner_setting_implementati___4 | Organizati incentives rewards (extrinsic incentives, bonuses, increased stature)<br>5 | inner_setting_implementati___5 | Goals and feedback (well goals communic and fed ba staff)<br>6 | inner_setting_implementati___6 | Learning c (organizati climate tha allows for reflective evaluation
10	[inner_setting_readiness_fo]	Inner Setting - Readiness for Implementation (BARRIERS)	checkbox<br>1 | inner_setting_readiness_fo___1 | Leadership engagemer (commitme involvemer and accountabi of leaders)<br>2 | inner_setting_readiness_fo___2 | Availability resources (dedicated resources f implementa and ongo n operations- money, training,

			education, time)<br>3 | inner_setting_readiness_fo___3 | Access to knowledge informatio (ease of acc to digestibl informatio and knowle about how works and incorporate into tasks)
11	[indchar_barrier]	Characteristics of Individuals (BARRIERS)	checkbox<br>1 | indchar_barrier___1 | Knowledge and beliefs about intervention (attitudes and values placed on the intervention)<br>2 | indchar_barrier___2 | Self-efficacy (abilities to execute intervention)<br>3 | indchar_barrier___3 | Individual stage of change (phase of change as progress toward sustained use of intervention)
12	[notes_facilitator]	Section Header: *Implementation Facilitators*<br>Implementation Facilitator Notes	notes
13	[intervention_facilitator]	Intervention Characteristics (FACILITATORS)	checkbox<br>1 | intervention_facilitator___1 | Intervention so (whether In Ou was internal/ex developed)<br>2 | intervention_facilitator___2 | Evidence, stren quality (whethe DNAS C was bu good evidence)<br>3 | intervention_facilitator___3 | Relative advant Our DNA SC is l than alternative<br>4 | intervention_facilitator___4 | Adaptability (In SC can be adapted/tailore discussions abo adaptations tha needed or mad<br>5 | intervention_facilitator___5 | Trialability (can Our DNA SC on scale)<br>6 | intervention_facilitator___6 | Complexity (ho radical, disrupti

						implementatio
				7	intervention_facilitator___7	Design, quality, packaging (exc how bundled, p assembled)
				8	intervention_facilitator___8	Cost (investme opportunity)
14	[outersetting_facilitator]	Outer Setting (FACILITATORS)	checkbox	1	outersetting_facilitator___1	Patient needs a resources (patients needs are accurately known and prioritized by organization)
				2	outersetting_facilitator___2	Cosmopolitanis (network with other external universities/hea systems)
				3	outersetting_facilitator___3	Peer pressure (pressure to implement program based other key peer organizations)
				4	outersetting_facilitator___4	External policie and incentives (policy and regulatory mandates, collaboratives)
15	[innersetting_facilitator]	Inner Setting (FACILITATORS)	checkbox	1	innersetting_facilitator___1	Structural characteristics (age, size, maturity of organization)
				2	innersetting_facilitator___2	Networks and communicatio
				3	innersetting_facilitator___3	Culture (norms, values, basic assumptions)
16	[inner_setting_imp_fac]	Inner Setting- Implementation Climate (FACILITATORS)	checkbox	1	inner_setting_imp_fac___1	Tension for change (current situation needs changed- can be about need to change anytime in the life course of implementatio
				2	inner_setting_imp_fac___2	Compatibility (fi between

			<table><tr><td></td><td></td><td>intervention and existing systems)</td></tr><tr><td>3</td><td>inner_setting_imp_fac___3</td><td>Relative Priority (importance of implementation within organization)</td></tr><tr><td>4</td><td>inner_setting_imp_fac___4</td><td>Organizational incentives and rewards (extrinsic incentives, bonuses, increased stature)</td></tr><tr><td>5</td><td>inner_setting_imp_fac___5</td><td>Goals and feedback (how well goals are communicated and fed back to staff)</td></tr><tr><td>6</td><td>inner_setting_imp_fac___6</td><td>Learning climate (organizational climate that allows for reflective evaluation)</td></tr></table>
17	[implementation_climate_rea]	Implementation Climate- Readiness for Implementation (FACILITATORS)	checkbox<table><tr><td>1</td><td>implementation_climate_rea___1</td><td>Leadersh engagem (commitn involveme and accountal of leaders</td></tr><tr><td>2</td><td>implementation_climate_rea___2</td><td>Availabilit resources (dedicate resources implemen and ongo operation money, training, education time)</td></tr><tr><td>3</td><td>implementation_climate_rea___3</td><td>Access to knowledg informati (ease of a to digesti informati and know about ho works an</td></tr></table>

			incorpora<br>into tasks
18	[indchar_facilitator]	Characteristics of Individuals (FACILITATOR)	checkbox<br>1 | indchar_facilitator___1 | Knowledge and beliefs about intervention (attitudes and values placed on the intervention)<br>2 | indchar_facilitator___2 | Self-efficacy (abilities to execute intervention)<br>3 | indchar_facilitator___3 | Individual stage of change (phase of change as progress toward sustained use of intervention)
19	[process]	Section Header: *Additional Details*<br>What phase of PROCESS is this?	checkbox<br>1 | process___1 | Planning (developing in advance, efforts to promote intervention collectively and individually)<br>2 | process___2 | Engaging (engaging members of teams tasked with implementing an intervention)<br>3 | process___3 | Executing (carrying out or accomplishing implementation according to the plan)<br>4 | process___4 | Reflecting and Evaluating (evaluation of implementation efforts)
20	[other_notes]	Other Notes	notes
21	[relevant_files]	Relevant File 1	file
22	[relevant_file_2]	Relevant File 2	file
23	[relevant_file_3]	Relevant File 3	file
24	[work_group_meeting_logs_complete]	Section Header: *Form Status*<br>Complete?	dropdown<br>0 | Incomplete<br>1 | Unverified<br>2 | Complete
